# Application of hemostatic forceps to treat post-sphincterotomy bleeding near the pancreatic duct opening

**DOI:** 10.1055/a-2353-5910

**Published:** 2024-07-26

**Authors:** Jay A. Bapaye, Reid D. Wasserman, Klaus Mönkemüller, Vivek Kesar, Varun Kesar

**Affiliations:** 1 6912 Gastroenterology, Carilion Clinic, Roanoke, United States; 2 6912 Internal Medicine, Carilion Clinic, Roanoke, United States; 3 Carilion School of Medicine, Virginia Tech, Roanoke, United States

A 36-year-old man underwent endoscopic retrograde cholangiopancreatography (ERCP) with sphincterotomy, which resulted in the extraction of several large stones. A 10-mm × 4-cm fully covered metal stent was placed in the bile duct and a 5-Fr × 4-cm flapless straight plastic stent in the pancreatic duct. The patient returned to the emergency department 2 days later complaining of dizziness, melanic stool, and a syncopal event. His hemoglobin showed a downward trend of 16 to 8.3g/dL. The patient underwent esophagogastroduodenoscopy, which showed active oozing with an adherent clot at the sphincterotomy site at the inferior aspect of the major papilla ( Fig. 1 ). The biliary stent was removed for better visualization, the clot was suctioned, and epinephrine (1:10 000) was injected around the site. Next, a hemostatic forceps (coagrasper) was utilized to inspect and expose the area ( Fig. 2 ). Blood was seen to be issuing near the pancreatic duct as well as a visible vessel at the apex of the papilla ( Video 1 ). Because of the tight space and the risk of clipping the pancreatic duct, the coagrasper was used to achieve hemostasis. Before applying coagulation, the ampulla was submerged under water to decrease the risk of thermal burn to the thin duodenal wall ( Fig. 3 ). A 10-mm × 4-cm biliary stent was deployed, and a 5-Fr × 4-cm stent was placed in the pancreatic duct to prevent post-procedure pancreatitis.

**Fig. 1:**
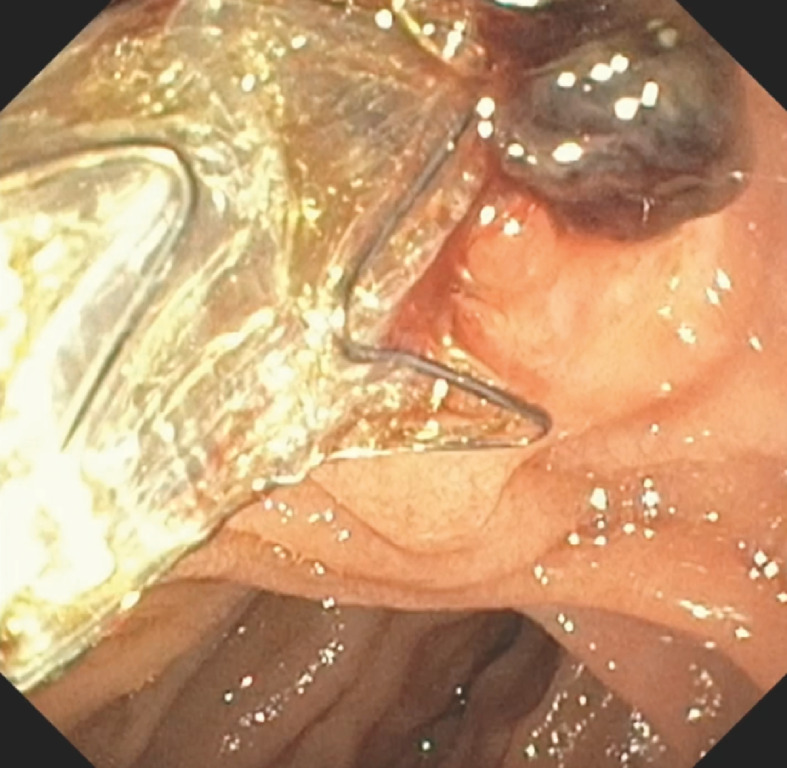
Active oozing with a blood clot at the inferior aspect of the major papilla, 2 days after endoscopic retrograde cholangiopancreatography (ERCP) with sphincterotomy.

**Fig. 2:**
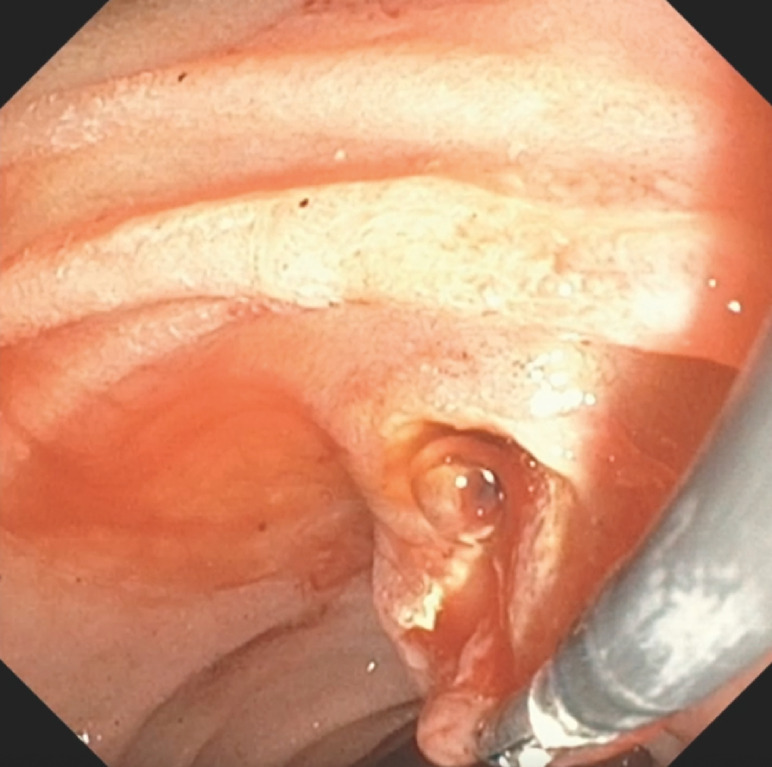
The hemostatic forceps (coagrasper) was used to pull the duodenal tissue toward the lumen in order to expose and identify the site of the bleeding.

**Fig. 3:**
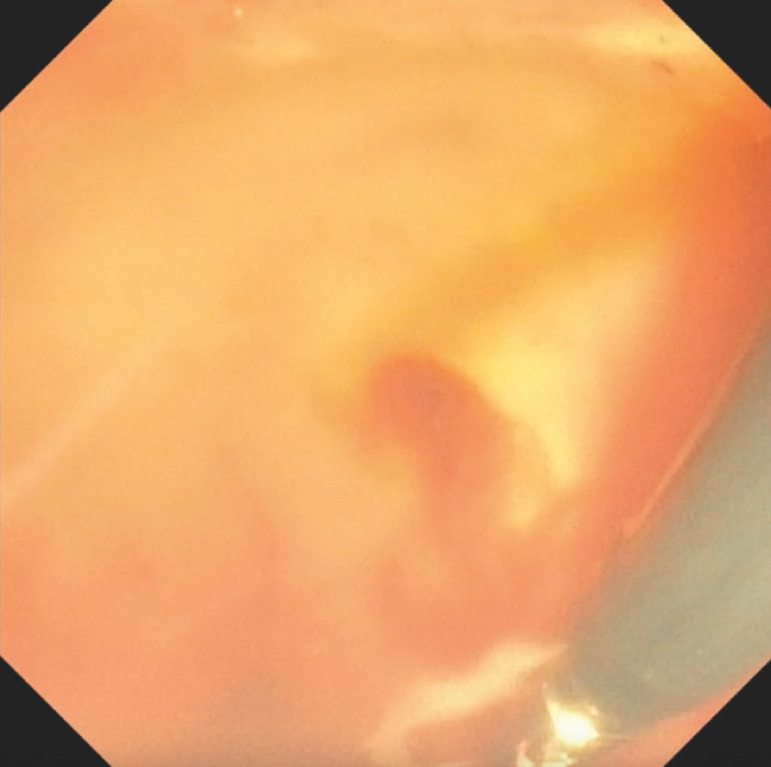
Monopolar coagulation using a coagrasper at the post-sphincterotomy site while submerged underwater.

Post-sphincterotomy bleeding treated with a monopolar hemostatic forceps (coagrasper) with underwater submersion.Video 1

Sphincterotomy is commonly performed to facilitate stone removal from the bile duct during ERCP, but does not come without complications such as bleeding. Bleeding may occur immediately or may be delayed, and one study reported a bleeding rate up to 12.1% 1 . Epinephrine injection is an effective and safe treatment modality for post-sphincterotomy bleeding, with two large studies showing success rates of 97.5% and 100%, respectively 1 2 . Hemostatic forceps are another tool for achieving hemostasis and are commonly used in endoscopic submucosal dissection. They have been shown to be successful for post-sphincterotomy bleeding in few case studies with a success rate of up to 100% 3 4 . The present case shows successful hemostasis with epinephrine and hemostatic forceps in a patient with post-sphincterotomy bleeding.

Endoscopy_UCTN_Code_CPL_1AK_2AC

## Notes

Endoscopy E-Videos https://eref.thieme.de/e-videos
*E-Videos* is an open access online section of the journal *Endoscopy* , reporting on interesting cases and new techniques in gastroenterological endoscopy. All papers include a high-quality video and are published with a Creative Commons CC-BY license. Endoscopy E-Videos qualify for HINARI discounts and waivers and eligibility is automatically checked during the submission process. We grant 100% waivers to articles whose corresponding authors are based in Group A countries and 50% waivers to those who are based in Group B countries as classified by Research4Life (see: https://www.research4life.org/access/eligibility/ ). This section has its own submission website at https://mc.manuscriptcentral.com/e-videos .
